# Abrogated Caveolin-1 expression via histone modification enzyme Setdb2 regulates brain edema in a mouse model of influenza-associated encephalopathy

**DOI:** 10.1038/s41598-018-36489-8

**Published:** 2019-01-22

**Authors:** Natsuko Imakita, Masahiro Kitabatake, Noriko Ouji-Sageshima, Atsushi Hara, Shoko Morita-Takemura, Kei Kasahara, Akihiro Matsukawa, Akio Wanaka, Keiichi Mikasa, Toshihiro Ito

**Affiliations:** 1 Department of Immunology, Nara Medical University, Kashihara, Nara Japan; 2 Center for Infectious Diseases, Nara Medical University, Kashihara, Nara Japan; 3 Department of Anatomy & Neuroscience, Nara Medical University, Kashihara, Nara Japan; 4 Department of Pathology and Experimental Medicine, Graduate School of Medicine, Dentistry and Pharmaceutical Sciences, Okayama University, Okayama, Japan

## Abstract

Influenza-associated encephalopathy (IAE) is a serious complication that can follow influenza virus infection. Once a cytokine storm is induced during influenza virus infection, tight junction protein disruption occurs, which consequently leads to blood-brain barrier (BBB) breakdown. However, the details of IAE pathogenesis are not well understood. Here, we established a murine IAE model by administration of lipopolysaccharide following influenza virus infection. Brains from IAE model mice had significantly higher expression of type I interferons and inflammatory cytokines. In addition, the expression of Caveolin*-*1, one of the key proteins that correlate with protection of the BBB, was significantly lower in brains from the IAE group compared with the control group. We also found that, among 84 different histone modification enzymes, only SET domain bifurcated 2 (Setdb2), one of the histone methyltransferases that methylates the lysine 9 of histone H3, showed significantly higher expression in the IAE group compared with the control group. Furthermore, chromatin immunoprecipitation revealed that methylation of histone H3 lysine 9 was correlated with repression of the *Caveolin-1* promoter region. These studies identify Caveolin-1 as a key regulator of BBB permeability in IAE and reveal that it acts through histone modification induced by Setdb2.

## Introduction

Influenza-associated encephalopathy (IAE) is a syndrome with acute impaired consciousness following influenza virus infection. IAE mainly occurs in children, especially those under 10 years old, and its mortality and sequelae rates are high^1,2^. Viremia is rarely seen in patients with IAE, and virus antigens have not been detected in cerebrospinal fluid samples from these patients^3–5^. Additionally, although brain autopsies from IAE cases have revealed brain edema, few inflammatory cell infiltration, and no virus antigens were observed^3,5–7^. Despite the lack of virus antigens, levels of inflammatory cytokines, such as tumor necrosis factor (TNF)-α, interleukin (IL)-6, and IL-1β, are elevated in the serum and cerebrospinal fluid of IAE patients, and high levels of these cytokines are related to the clinical severity of the disease^8^. Elevation of these inflammatory cytokines, called a “cytokine storm”, is thought to be responsible for IAE pathogenesis^3,8–15^. The cytokine storm causes a disruption of tight junction proteins, leading to blood-brain barrier (BBB) breakdown^16^, and this is considered to be associated with the mechanism of IAE induction.

The BBB is mainly formed by brain capillary endothelial cells, pericytes, astrocytes, and neuronal cells^17^. It plays a major role in maintaining cerebral homeostasis by acting as a barrier that separates the central nervous system from the peripheral blood circulation. The BBB also regulates the movement of ions, oxygen, and nutrients between the blood and brain and restricts the invasion of toxins and pathogens^18^. Inflammatory cytokines, such as TNF-α, IL-1β, and IL-6, activate endothelial cells in the BBB, and the overproduction of these cytokines, i.e. a “cytokine storm”, leads to the disruption of homeostasis and an increase in permeability^19^. Furthermore, many studies have recently suggested that the cytokine storm also induces epigenetic changes, such as histone modification of various promoter regions, in models of sepsis and severe infection^20–25^. However, the details concerning the mechanism of IAE pathogenesis remain unclear, and an effective specific treatment for IAE has not been established.

Lipopolysaccharide (LPS) is a major component of the outer membrane of Gram-negative bacteria, and it is known to induce inflammatory cytokines, such as TNF-α, IL-6, IL-1β, type I interferons (IFN-α, IFN-β)^26,27^, and type II IFN (IFN-γ)^28^. There have not been reported relevant IAE models using single influenza virus infection. Although there are several publications that describe IAE-like mouse models using LPS administration in addition to influenza virus infection^29,30^, the routes, doses, and timings of LPS administration differ between these studies. It is still debated whether endotoxemia in influenza virus infection associates with the pathogenesis of IAE, but the IAE-like mouse model shows hypercytokinemia without direct influenza virus infection in the brain, and that is closely intimate histological and immunopathological features such as BBB breakdown seen in IAE patients. Therefore, IAE-like mouse model is considered a useful tool for the better understanding of IAE pathogenesis^31^.

Here, we developed an IAE mouse model by intravenously administering LPS following influenza virus infection. Using this IAE model, we focused on the tight junction proteins in the BBB and investigated the regulation of tight junction proteins induced by histone modification.

## Results

### Establishment of a murine IAE model

**Figure 1:**
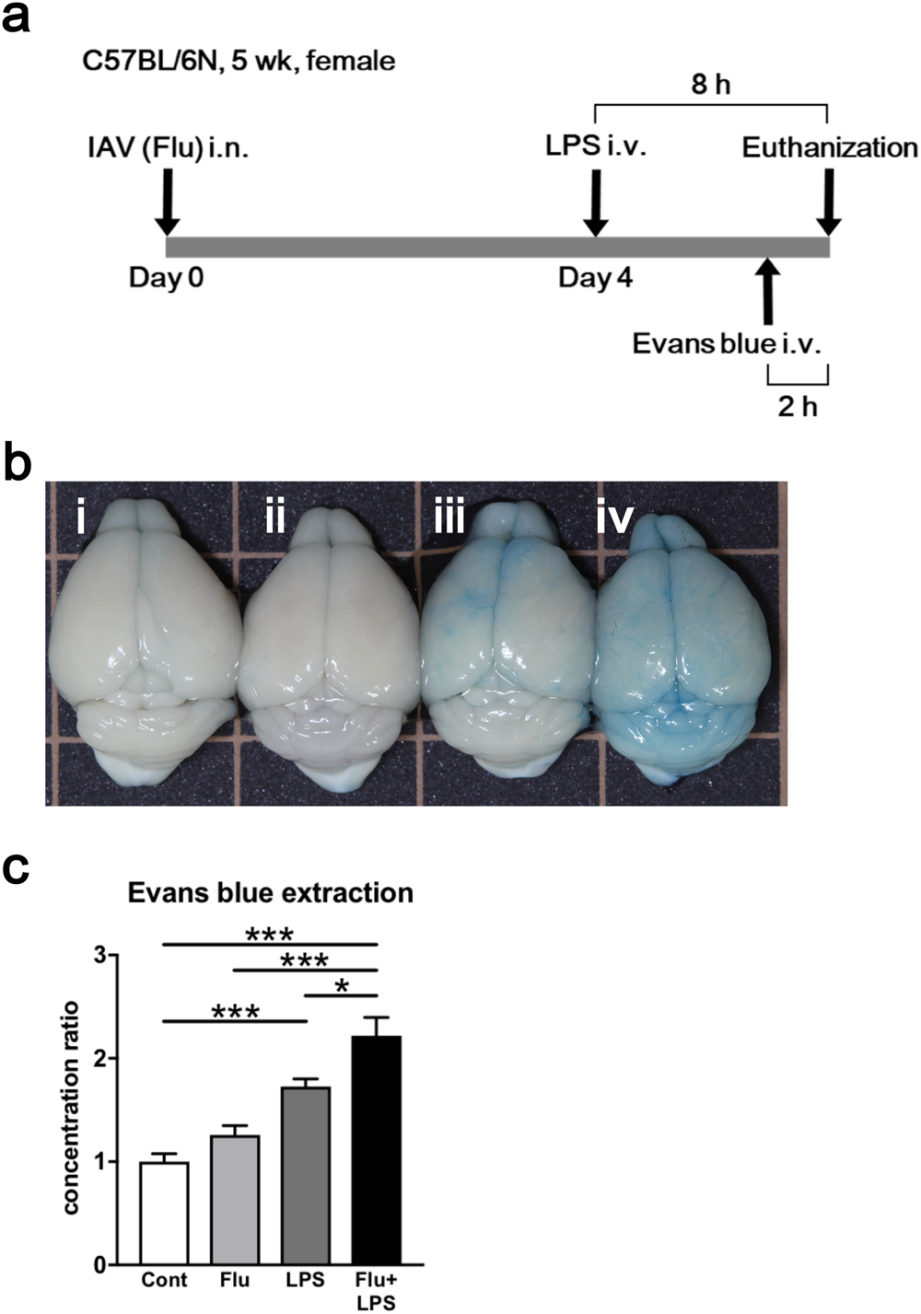
Evaluation of brain edema caused by influenza associated encephalopathy (IAE) model using Evans blue administration. (**a**) Time course schematic of the murine IAE model. Four days after influenza virus (IAV) intranasal inoculation (3 × 10^5^ pfu), mice were intravenously administered 40 mg/kg LPS. Eight hours after LPS administration, the mice were analysed. In some experiments, Evans blue was intravenously injected into mice 2 h before analysis. **b**) Macroscopic appearance of the brains from the (i) control, (ii) Flu, (iii) LPS, and (iv) IAE (Flu + LPS) groups. (**c**) Ratios of the concentration of extracted Evans blue dye from the brains of various groups to those from control mice. Data are shown as the mean ± SEM (control, *n* = 7; Flu, *n* = 8; LPS, *n* = 12; Flu + LPS, *n* = 8) and are from a representative experiment of three independent experiments. *p < 0.05; ***p < 0.001.

We first tried to establish a murine model of IAE using only influenza A virus (IAV) infection. However, the intranasal inoculation of IAV alone was not enough to induce IAE, even when the dose of IAV was increased to a lethal dose (data not shown). We next added the intravenous administration of LPS following IAV infection (Flu) to our model, as described in Fig. 1a. To evaluate whether IAE was induced or not, we examined the occurrence of brain edema by intravenously administering Evans blue dye 2 h before euthanization. In the presence of Evans blue dye, brain edema is stained blue, and its appearance suggests that the BBB was broken, confirming the establishment of IAE.

After Evans blue dye administration, brains from the control and Flu groups were not blue, brains from the LPS group turned partially blue, and brains from the Flu + LPS group turned completely blue (Fig. 1b). Next, we quantified this difference by measuring the amount of Evans blue dye extracted from these whole brains. The Evans blue dye concentration of the control group was set at 1. While the concentration ratio of the LPS group was significantly higher compared with that of the control groups, that of the Flu + LPS group was more increased and significantly higher compared with not only that of the control and Flu group but also that of the LPS group (Fig. 1c). These results demonstrate that additional factors such as LPS are essential to induce IAE, and that a murine model using LPS administration following IAV infection can be defined as an IAE model.

### Strong cytokine induction in whole brains from IAE model mice

**Figure 2:**
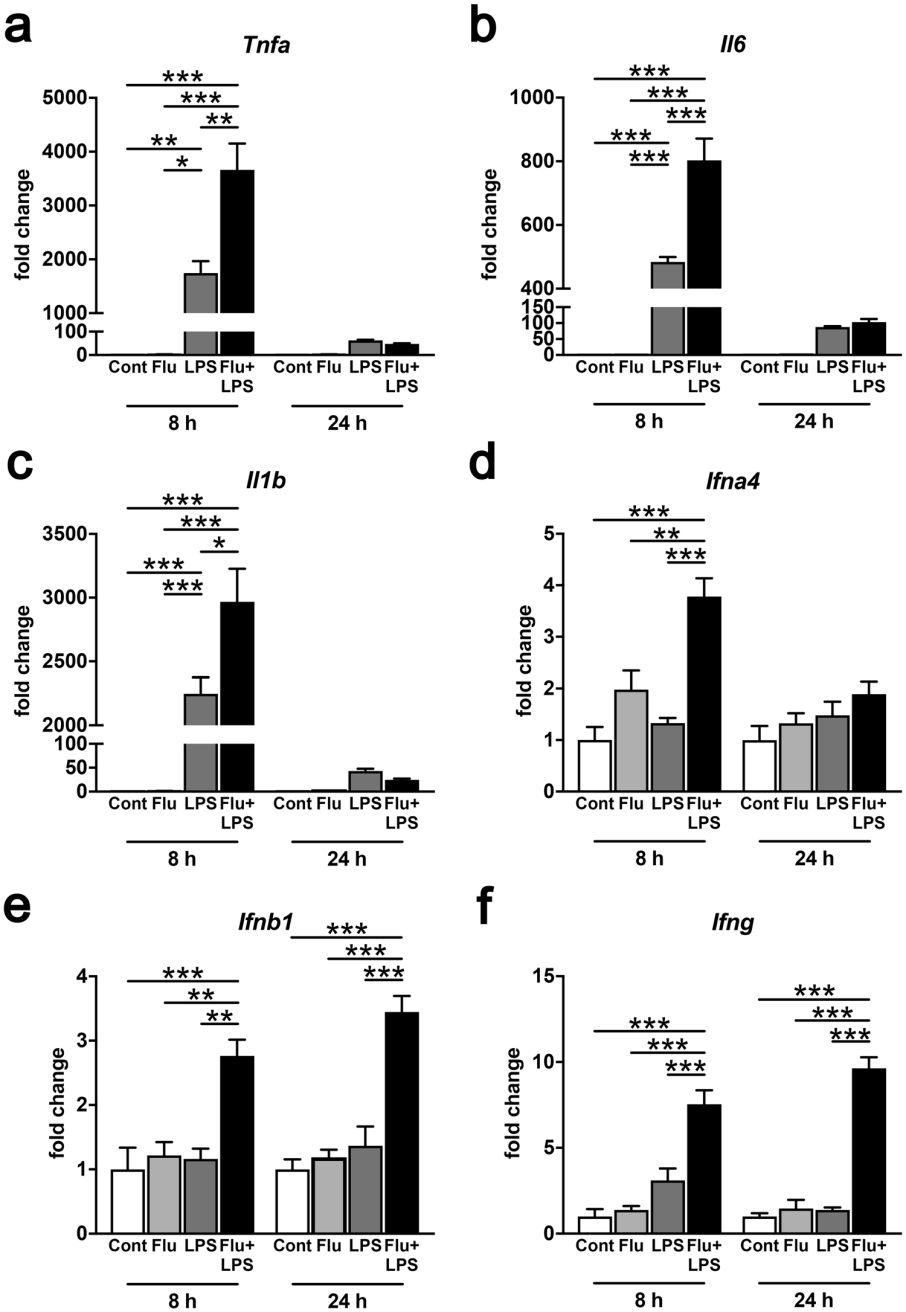
Levels of inflammatory cytokines and IFNs in IAE model mice. Gene expressions of *Tnfa* (**a**), *Il6* (**b**), *Il1b* (**c**), *Ifna4* (**d**), *Ifnb1* (**e**), and *Ifng* (**f**) from whole brains at 8 h and 24 h post LPS administration were analysed by quantitative PCR. Data are shown as the mean ± SEM (8 h control, *n* = 5; Flu, *n* = 3; LPS, *n* = 5; Flu + LPS, *n* = 5; 24 h control, *n* = 5; Flu, *n* = 5; LPS, *n* = 3; Flu + LPS, *n* = 6) and are from a representative experiment of two or three independent experiments. *p < 0.05; **p < 0.01; ***p < 0.001.

To help elucidate the mechanism underlying the changes in the brains of IAE cases, we profiled the cytokine expression in the brains of IAE model mice. We observed that whole brains from the LPS group had significantly higher mRNA expression levels of *Tnfa*, *Il6*, and *Il1b* in comparison with those from the control and Flu groups at 8 h post LPS administration, and the levels of these inflammatory cytokines in whole brains from the IAE (Flu + LPS) group were even higher than those from the LPS group (Fig. 2a–c). We also measured the expression levels of type I IFNs and type II IFN (IFN-γ). Although both type I IFNs and type II IFN were expressed at detectable levels in brains from the control, Flu, and LPS groups, brains from the IAE group expressed significantly higher levels of *Ifna4*, *Ifnb1* and *Ifng* at 8 h post LPS administration. Moreover, gene expressions of *Ifnb1* and *Ifng* were significantly higher in the IAE group even at 24 h post LPS administration (Fig. 2d–f).

### Lower tight junction protein expression in whole brains from IAE model mice

**Figure 3:**
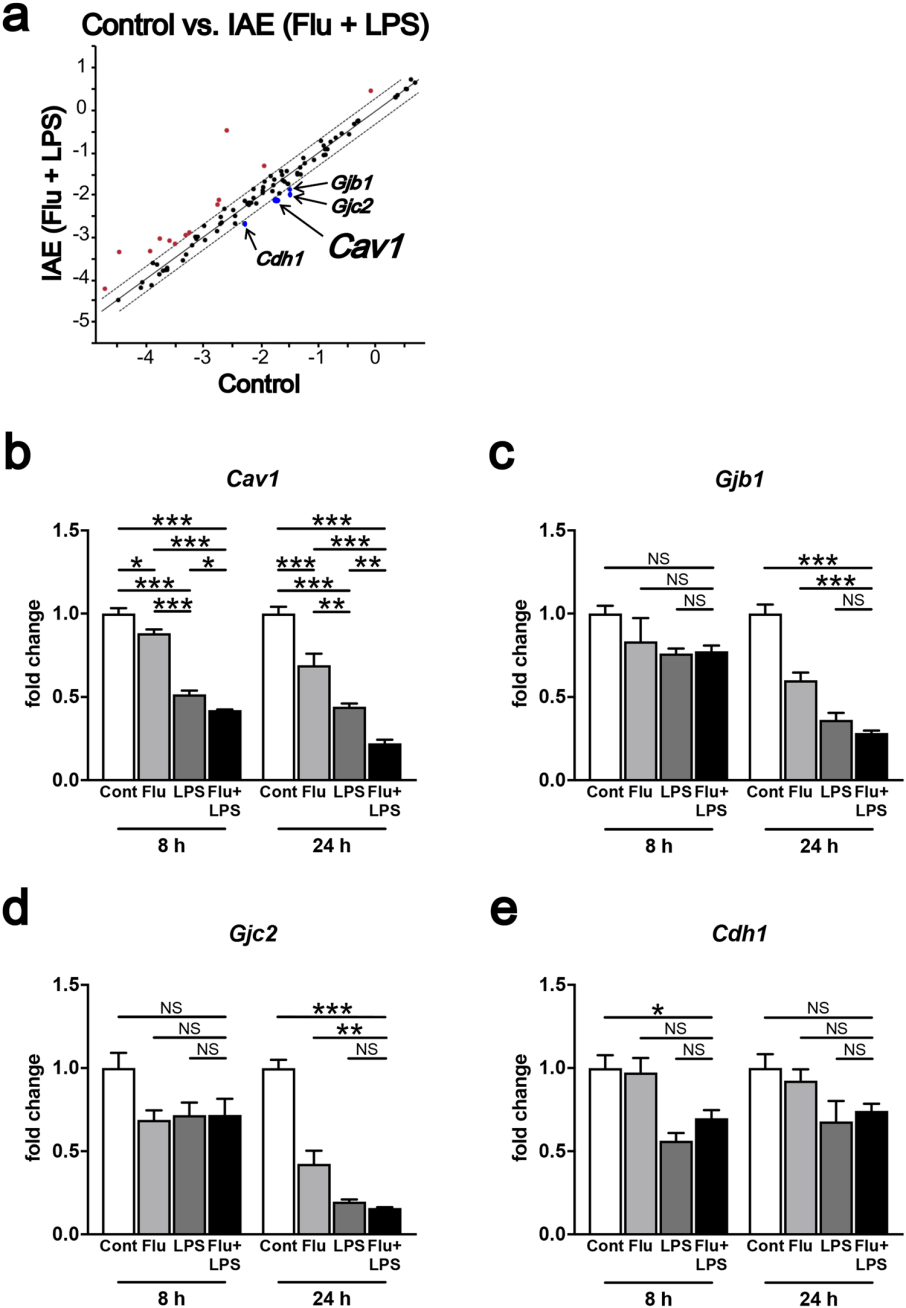
Gene expression of tight junction proteins in whole brains from IAE model mice. (**a**) The gene expressions of 84 cell junction proteins in whole brains from the control and IAE (Flu + LPS) groups at 8 h post LPS administration were analysed by PCR array. The fold regulation threshold was 2. (**b**–**e**) The expression levels of *Cav1* (**b**), *Gjb1* (**c**), *Gjc2* (**d**), and *Cdh1* (**e**) in whole brains at 8 h and 24 h post LPS administration were analysed. Data are shown as the mean ± SEM (8 h control, *n* = 5; Flu, *n* = 3; LPS, *n* = 5; Flu + LPS, *n* = 5; 24 h control, *n* = 5; Flu, *n* = 5; LPS, *n* = 3; Flu + LPS, *n* = 6) and are from a representative experiment of two or three independent experiments. NS; not significant. *p < 0.05; **p < 0.01; ***p < 0.001.

BBB breakdown in IAE is thought to be caused by tight junction disruption following a cytokine storm^16^. First, to directly define which cell junction-related protein is suppressed in our IAE model, we performed a polymerase chain reaction (PCR) array for proteins related to tight junctions, gap junctions, adherens junctions, focal adhesions, desmosomes, and hemidesmosomes, using whole brain samples. Of the 84 tested genes for cell junction-related proteins, the expressions of *Caveolin-1* (*Cav1*), *Gap junction β-1* (*Gjb1*), *Gap junction γ-2* (*Gjc2*), and *Cadherin-1* (*Cdh1*) were all significantly lower in the IAE group compared with the control group (Fig. 3a). A follow-up quantitative PCR of these four genes revealed that *Cav1* was the only molecule whose gene expression was significantly lower in the IAE group compared with the control, Flu, and LPS groups (Fig. 3b–e).

### Impaired Caveolin-1 level in brain vascular endothelial cells from IAE model mice

**Figure 4:**
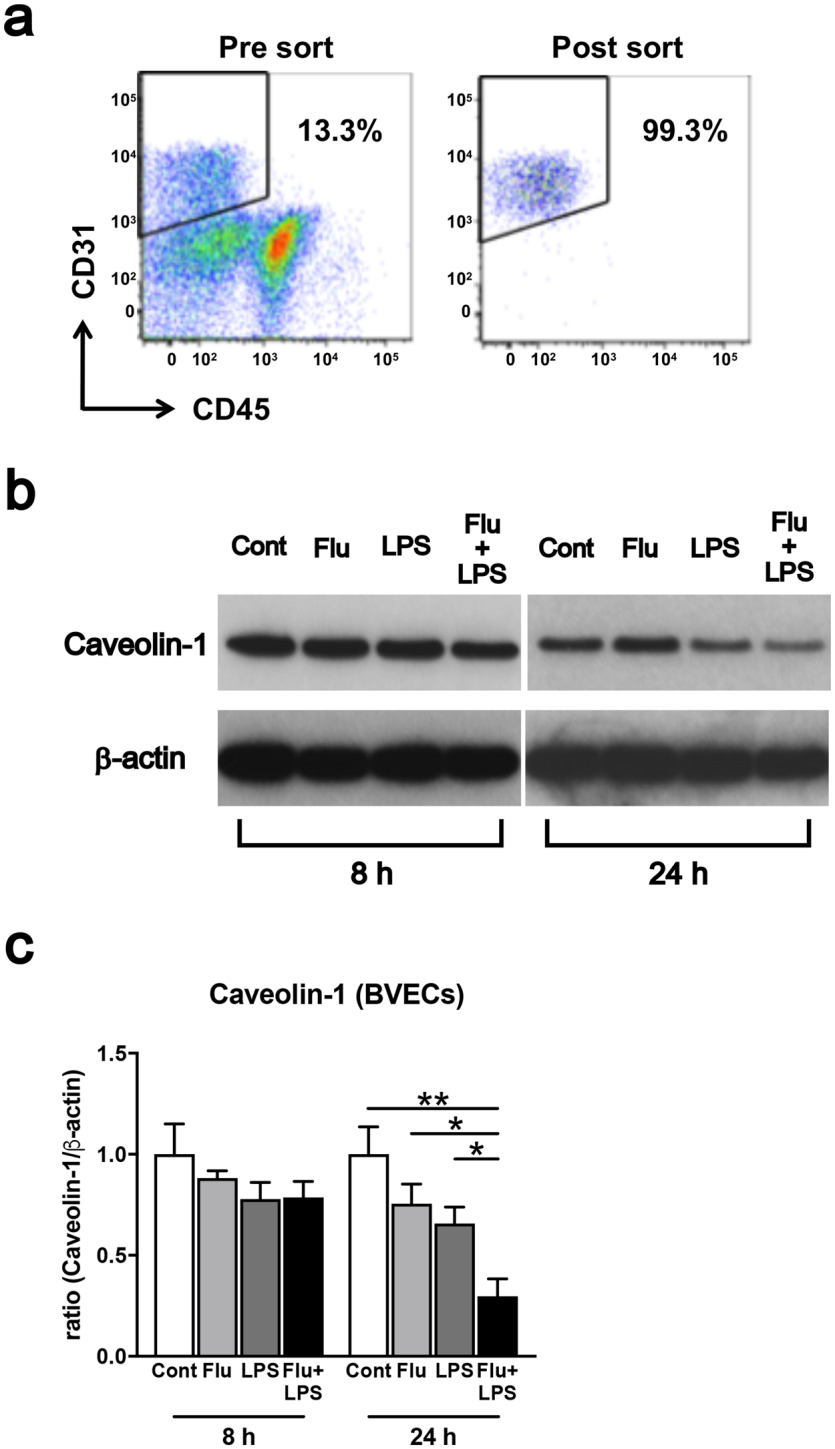
Caveolin-1 level in brain vascular endothelial cells (BVECs). (**a**) Flow cytometry was performed using specific antibodies against CD31 and CD45. Sorted CD31^+^CD45^−^ cells were defined as BVECs. The purity of this group was >98%. (**b**) Western blot analysis of Caveolin-1 and β-actin from BVECs of the control, Flu, LPS and IAE (Flu + LPS) groups. Analysis was performed 8 h and 24 h after LPS administration. (**c**) The bands of Caveolin-1 and β–actin were quantified using Image J. Data are shown as the mean ± SEM (8 h control, *n* = 4; Flu, *n* = 4; LPS, *n* = 4; Flu + LPS, *n* = 4; 24 h control, *n* = 4; Flu, *n* = 4; LPS, *n* = 4; Flu + LPS, *n* = 4) and are from a representative experiment of two independent experiments. *p < 0.05; **p < 0.01.

To investigate whether the relatively lower *Cav1* expression in whole brains from IAE model mice can be attributed to vascular endothelial cells, we sorted out the subset of CD31^+^CD45^−^ cells, which we defined as brain vascular endothelial cells (BVECs), from whole brains and then analysed the Caveolin-1 level in the BVECs. The purity of the sorted CD31^+^CD45^−^ cells was >98% (Fig. 4a). In western blot analysis, the Caveolin-1 level in the BVECs was slightly lower at 8 h after LPS administration in the LPS and IAE groups, while the Caveolin-1 level at 24 h after LPS administration was also significantly lower in the IAE group compared with the control, Flu and LPS groups (Fig. 4b,c, Supplementary Fig. S1).

### Induction of histone modification enzymes in IAE model mice

**Figure 5:**
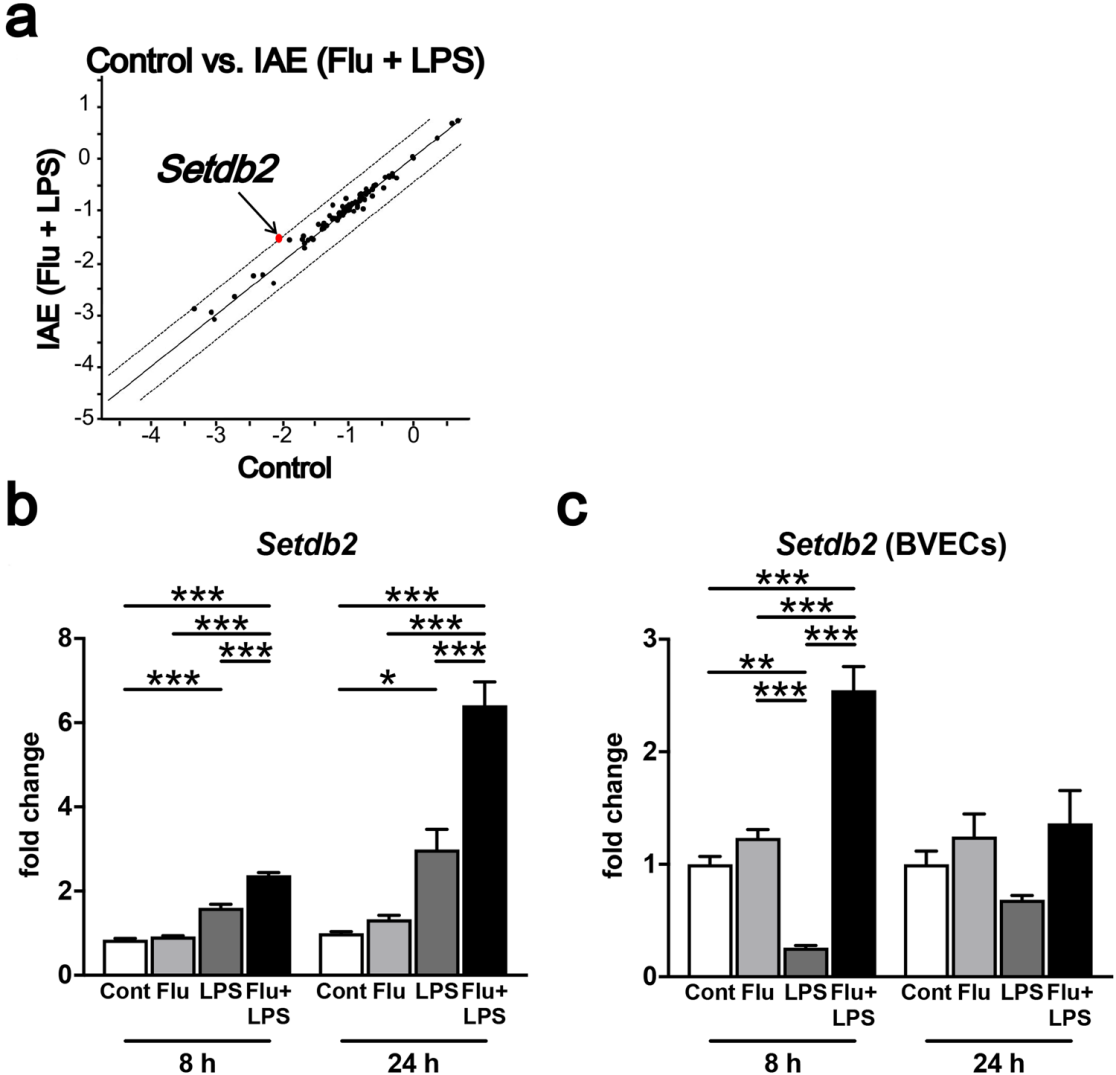
*Setdb2* expression in IAE model mice. (**a**) The gene expressions of 84 epigenetic chromatin modification enzymes in whole brains from the IAE group at 8 h post LPS administration were analysed by PCR array. The fold regulation threshold was 3. (**b**) Gene expression of *Setdb2* in whole brains at 8 h and 24 h post LPS administration. Data are shown as the mean ± SEM (8 h control, *n* = 5; Flu, *n* = 3; LPS, *n* = 5; Flu + LPS, *n* = 5; 24 h control, *n* = 5; Flu, *n* = 5; LPS, *n* = 3; Flu + LPS, *n* = 6) and are from a representative experiment of two or three independent experiments. *p < 0.05; ***p < 0.001. (**c**) Gene expression of *Setdb2* at 8 h and 24 h post LPS administration in BVECs. Data are shown as the mean ± SEM (8 h control, *n* = 4; Flu, *n* = 4; LPS, *n* = 4; Flu + LPS, *n* = 4; 24 h control, *n* = 3; Flu, *n* = 3; LPS, *n* = 3; Flu + LPS, *n* = 3) and are from a representative experiment of two or three independent experiments. **p < 0.01; ***p < 0.001.

From the above results, we suspected that a cytokine storm can induce the expression of histone-modifying enzymes, which, in turn, regulate the Caveolin-1 level in BVECs. To determine which histone-modifying enzymes are regulated during IAE pathogenesis, we next performed a PCR array for genes of chromatin modification enzymes. Of the 84 tested genes for chromatin modification enzymes, only SET domain bifurcated 2 (*Setdb2*) had a significantly higher expression level in the IAE group compared with the control group (Fig. 5a). The *Setdb2* expression from whole brains was significantly higher at both 8 h and 24 h following LPS administration in IAE group (Fig. 5b). On the other hand, Setdb2 expression in BVECs from LPS group was remarkably decreased, while that from IAE group was significantly increased compared with control, Flu, and LPS groups at 8 h following LPS administration (Fig. 5c).

### H3K9 methylation in the *Cav1* promoter region was higher in IAE model mice

**Figure 6:**
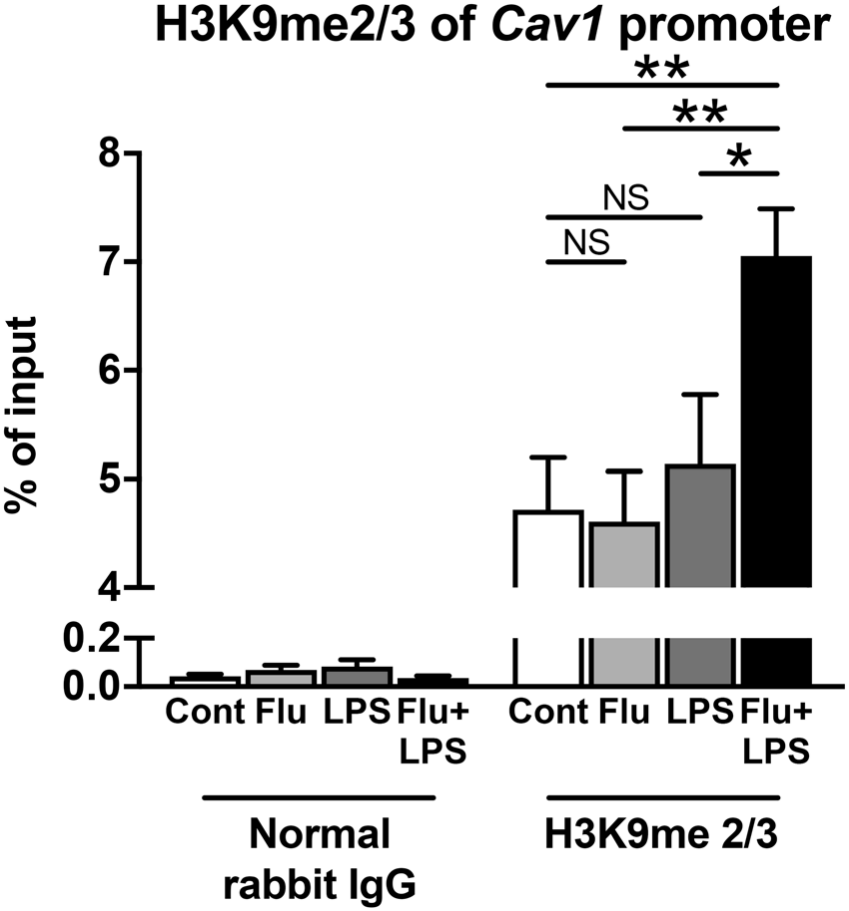
H3K9 methylation at the *Cav1* promoter region in IAE model mice. The H3K9 methylation levels at the promoter of the *Cav1* gene in whole brains from the control, Flu, LPS, and IAE groups at 8 h post LPS administration were assessed by chromatin immunoprecipitation (ChIP). Combined data are shown as the mean ± SEM (control, *n* = 8; Flu, *n* = 4; LPS, *n* = 4; Flu + LPS, *n* = 7). NS; not significant. *p < 0.05; **p < 0.01.

Setdb2 is one of the histone methyltransferases that induces suppression of target gene expression by methylating the lysine 9 of histone H3 (H3K9)^32^. We next used chromatin immunoprecipitation (ChIP) to examine whether H3K9 methylation is correlated with suppression of *Cav1* gene expression. ChIP analysis revealed that H3K9 methylation was significantly higher in the *Cav1* promoter region of whole brains in IAE group compared with the control, Flu, and LPS groups (Fig. 6).

## Discussion

IAE is a serious potential complication following influenza virus infection. Previous clinical reports indicate that the levels of TNF-α, IL-6, and IL-1β are elevated in serum and cerebrospinal fluid samples from patients with IAE^3,8–15^, revealing the presence of a cytokine storm. Notably, cytokine storms are correlated with various kinds of infectious and non-infectious diseases, and they often lead to unfortunate outcomes. Aberrant cytokine production had been correlated with the expression of tight junction proteins; for example, TNF-α decreases occludin and claudin-5 expression^33^, and IL-1β reduces occludin, claudin-5, and ZO-1 expression^34^. IFN-γ is also reported that it enhances BBB permeability by down-regulating and internalizing tight junction proteins^35,36^. The cytokine storm following influenza virus infection is thought to be one of the critical causes of IAE, acting via disruption of tight junctions. The BBB breakdown following tight junction disruption is the key mechanism of IAE pathogenesis^16^; however, the detailed mechanism for this process is still not understood.

As IAV infection alone was insufficient to induce IAE in a mouse model, we needed to apply an additional procedure to induce a cytokine storm. Some previous studies have used murine IAE models, but their models varied in terms of the mouse age, influenza virus type, inoculation route, and infection time course^29,30,37,38^. Several models have included LPS, which is a major component of the outer membrane of Gram-negative bacteria and is a ligand for Toll-like receptor 4. LPS is also known to induce inflammatory cytokines, such as TNF-α, IL-6, IL-1β, and type I IFNs by activating signal pathways through NF-κB and IFN regulatory factors (IRF). While systemic administration of LPS causes sepsis, including septic encephalopathy, which is pathologically similar to IAE in terms of the involvement of inflammatory cytokines including TNF-α, IL-6, IL-1β, type I IFNs^26,27,39^, and type II IFN^35,36^, our study clearly demonstrates that influenza virus infection prior to systemic administration of LPS exacerbated brain edema. We tried to make murine IAE model following previous publications^29,30,37,38^ including LPS dose selection, but the reproducibility was low in our experiment. Thus, we here established a murine model for IAE using 40 mg/kg LPS intravenous administration with 100% reproducibility and with significantly higher expression of type I IFNs and inflammatory cytokines in brain. Using this IAE model, we confirmed that the expression of histone methyltransferase *Setdb2* was relatively higher in the brains of IAE model mice. We also demonstrated that methylation of H3K9 by Setdb2 induction was associated with a significant reduction of Caveolin-1.

IAE is associated with severe neurologic sequelae. The acute encephalitis or encephalopathy in IAE is induced following influenza virus infection of the respiratory tract^16^. Recently, we and others have reported that epigenetic changes are induced in models of sepsis and severe infection^20–25^. In an influenza virus infection model, we found an increase of *Setdb2* expression, which is dependent on type I IFNs^40^. Setdb2 regulates monocyte and macrophage-mediated immunity during influenza virus infection by inhibiting the expression of *Cxcl1*^41^ or *Ccl2*^40^. Setdb2 methylates H3K9, and methylation of H3K9 is associated with gene repression^32,42,43^. The induction of both *Setdb2* and type I IFNs was confirmed in the brains of our IAE model mice, which suggests that the higher expression levels of *Setdb2* in both whole brains and BVECs of IAE model mice might be due to the induction of type I IFNs brain expression. Interestingly, the expression of *Setdb2* in whole brains from LPS group was not as high as IAE group, but elevated compared to those from the control group. On the other hand, the expression of *Setdb2* in BVECs from LPS group was significantly lower than those from the control group while those from IAE group was significantly increased. These results indicate that the mechanism of brain edema induction regulated by Setdb2 might be different between LPS group and IAE group. Further investigation is needed to examine whether the incidence of IAE can be changed by regulating Setdb2 specifically in BVECs.

Given that BBB breakdown is caused by the disruption of tight junction proteins, we suspected that epigenetic change, such as H3K9 methylation, following influenza virus infection might have an influence on tight junction protein expression. Here, we found that the Caveolin-1 level was significantly lower in BVECs from the IAE group compared with the control, Flu, and LPS groups. Caveolin-1 is the 21–24 kDa protein that forms caveolae, and although it is expressed in various cell types, such as smooth muscle cells, fibroblasts, adipocytes, epithelial cells, and endothelial cells^44^, it is predominately expressed in vessels in the central nervous system^45^. Caveolin-1 knockout mice display BBB hyperpermeability^46^. Additionally, a Caveolin-1 knockdown remarkably decreased the expression of one of the tight junction proteins, ZO-1, in BVECs^47^. In a cerebral ischemia injury model, Caveolin-1 deficiency also mediated BBB leakage^48,49^. It has been reported that Caveolin-1 acts as a mediator of anti-inflammatory effects by inhibiting inflammatory cytokine expression through NF-κB and AP-1^50^. However, few studies have examined the potential correlation between Caveolin-1 and inflammatory cytokines in BVECs. Furthermore, there were no reports about the potential correlation between Caveolin-1-mediated inflammatory cytokine regulation and the disruption of tight junction proteins forming the BBB.

These prior findings indicate that Caveolin-1 plays an important role in the regulation of endothelial function and that the loss of Caveolin-1 in BVECs induces BBB hyperpermeability. Our results further suggest that the relatively low Caveolin-1 level in our IAE model might be correlated with the IAE pathogenesis induced by BBB breakdown. In addition, our ChIP results show that H3K9 methylation was higher in the *Cav1* promoter region. Because Setdb2 methylates H3K9, and this H3K9 methylation represses the expression of target genes^32,42,43^, we suspect that H3K9 methylation by Setdb2 might repress the Caveolin-1 level in BVECs.

Additional studies are needed to examine which transcription factor binds to the *Cav1* promoter region and to investigate the pathway downstream from *Cav1* along with the potential correlations between Caveolin-1 and other tight junction proteins, such as ZO-1, claudins, and occludins. The present work does not address the question of how Caveolin-1 correlates with BBB permeability. However, previous reports revealed that TNF-α induces BBB breakdown by upregulating matrix metalloproteinases (MMPs) activity in *in vivo*^51^, and Caveolin-1 inhibits MMPs activation in both Caveolin-1 knockout mice and cerebral ischemic-reperfusion injury model animals^48,49,52^. Thus, Caveolin-1 might mediate MMPs activation in IAE by inhibiting the expressions of inflammatory cytokines; further investigation is necessary to test this possibility. Last, our data do not reveal the exact role of Caveolin-1 in IAE or how Caveolin-1 correlates with BBB permeability; the detailed mechanism by which epigenetic changes by Setdb2 regulate tight junctions in IAE pathogenesis remains to be elucidated.

In summary, this work provides a comprehensive analysis of epigenetic regulation of tight junctions in BVECs in an IAE mouse model. *Setdb2* expression was higher in the brains of IAE model mice, and the H3K9 methylation by Setdb2 induced a repression of the Caveolin-1. This led to BBB hyperpermeability and breakdown, which are both hallmarks of IAE pathogenesis. The present study supports the idea that an understanding of the relationship between epigenetic change, especially via Setdb2, and Caveolin-1 in BVECs during IAE can provide mechanistic approaches for controlling and modifying the BBB response during IAE.

## Methods

### Ethics statement

All animal experiments performed for this study were approved by The Animal Care and Use Committee at Nara Medical University (Approval No. 11367), and all methods were performed based on the Policy on the Care and Use of Laboratory Animals, Nara Medical University.

### Animal model for IAE

Five-week-old, female, specific pathogen-free C57BL/6N were purchased from CLEA Japan (Tokyo, Japan). The details of the Influenza A/Puerto Rico/8/34 (H1N1) used throughout the study were described previously^53^. For the IAE model, mice were anesthetized with isoflurane and intranasally (i.n.) administered influenza A virus (IAV, 3 × 10^5^ pfu) in 30 μl of saline (Otsuka Pharmaceutical Co., Ltd., Tokushima, Japan). Control mice were i.n. administered 30 μl of saline. Four days after infection, the mice were intravenously (i.v.) administered 40 mg/kg LPS (O55:B5) (Sigma-Aldrich, St. Louis, MO, USA) diluted in 200 μl of phosphate-buffered saline (PBS; Wako, Osaka, Japan) to generate the IAE model. IAV infection induced 10–20% body weight loss at Day 4, and the dosage of LPS was determined by the body weight at Day 4. Control mice were i.v. administered 200 μl of PBS. Eight and twenty-four hours after LPS administration, the mice were anesthetized with isoflurane followed by euthanization via blood removal with PBS perfusion. The mice were divided into four groups as follows: (i) Control group (saline i.n. + PBS i.v.), (ii) Flu group (IAV i.n. + PBS i.v.), (iii) LPS group (saline i.n. + LPS i.v.), and (iv) Flu + LPS group (IAV i.n. + LPS i.v.). All viral infections were performed in the P3 biosafety compartment of Nara Medical University.

### Evaluation of brain edema

To evaluate brain edema, the mice were i.v. administered with 10 μl/g of 2% Evans blue (Wako) 2 h before euthanization. The mice were anesthetized with isoflurane and perfused with PBS at 9 ml/min for 1 min. The brains were then removed and minced by scissors. The Evans blue in the minced brains was extracted with 500 μl of 4% paraformaldehyde for 2 days at 38 °C. After Evans blue extraction, the suspensions were centrifuged at 10000 × *g* for 20 min at room temperature, and the resulting supernatants were collected. The amounts of extracted Evans blue in these supernatants were measured by microplate reader (Thermo Fisher Scientific, Waltham, CA, USA) with a 620-nm wavelength filter against a standard of paraformaldehyde in PBS^30^.

### RNA extraction and quantitative PCR

Total RNA was extracted from brain halves using a NucleoSpin RNA kit (Macherey-Nagel GmbH & Co. KG., Düren, Germany). Brain tissues were homogenized with Zirconia beads and 700 μl of buffer RA1 including 1% 2-melcaptoethanol by a Beads Homogenizer (Wakenyaku Co., Ltd., Kyoto, Japan), after which 700 μl of 70% ethanol was added to each lysate. Half of the mixture was added to a NucleoSpin RNA Column and subsequently processed according to the manufacturer’s instructions. Extracted RNA was reverse transcribed with a SuperScript IV First-Strand Synthesis System (Thermo Fisher Scientific). Gene expressions were analysed with Taqman Fast Advanced Master Mix (Thermo Fisher Scientific) by quantitative PCR using Step One Plus (Applied Biosystems, Foster City, CA, USA). Taqman gene expression assays for *Gapdh* (Mm99999915), *Setdb2* (Mm01318753), *Cav1* (Mm00483057), *Gjb1* (Mm01950058), *Gjc2* (Mm00519131), *Cdh1* (Mm01247357), *Tnfa* (Mm00443258), *Il6* (Mm00446190), *Il1b* (Mm00434228), *Ifna4* (Mm00833969), *Ifnb1* (Mm00439552), and *Ifng* (Mm01168134) were purchased from Thermo Fisher Scientific. Relative quantitation was performed using the comparative CT (∆∆CT) method^53^. Data were normalized to *Gapdh* expression, as an endogenous control. ROX dye was used as the passive reference. The thermal cycling conditions were as follows: 95 °C for 20 s, followed by 40 cycles of amplification at 95 °C for 1 s and 60 °C for 20 s for denaturing and annealing.

### PCR array

The gene expressions of brains were analysed using an RT^2^ Profiler PCR Array Mouse Epigenetic Chromatin Modification Enzymes kit (Qiagen, Hilden, Germany) or an RT^2^ Profiler PCR Array Mouse Cell Junction PathwayFinder kit (Qiagen). Brain RNA was transcribed into complementary DNA (cDNA) by incubation with reverse transcriptase at 42 °C for 15 min followed by incubation at 95 °C for 5 min using an RT^2^ First Strand Kit (Qiagen). Complementary DNA was amplified with RT^2^ SYBR Green ROX qPCR Mastermix (Qiagen) according to the manufacturer’s instructions, and the gene expressions were normalized to multiple housekeeping genes. The results were analysed using RT^2^ Profiler PCR Array Data Analysis v3.5 (Qiagen), comparing the gene expression between the brains of the IAE and control groups.

### Brain vascular endothelial cell isolation

Brains were dissociated into single cell suspensions using a Neural Tissue Dissociation Kit (P) (Miltinyi Biotec GmbH, Cologne, Germany) with gentleMACS Dissociator. Myelins were removed from single cell suspensions using Myelin Removal Beads II (Miltinyi Biotec GmbH) and resuspended with 250 μl of 0.5% bovine serum albumin (BSA) in PBS following manufacturer’s instruction. For blocking Fc receptors, the cells were pre-incubated with purified anti-mouse CD16/32 antibody (BioLegend Inc., San Diego, USA) for 5 min. After incubation, the cells were washed and resuspend with 500 μl of 0.5% BSA in PBS. The cells were then incubated with phycoerythrin (PE)-labelled anti-CD31 monoclonal antibody (BioLegend Inc.) and allophycocyanin (APC)-labelled anti-CD45 monoclonal antibody (BioLegend Inc.) on ice for 30 min. Dead cells were excluded from brain cells using 7-Aminoactinomycin D (7-AAD, Becton, Dickinson & Co., Franklin Lakes, NJ, USA), and then CD31^+^CD45^−^ cells were isolated as BVECs using a FACSAria (Becton, Dickinson & Co.). The purity of BVECs was >98%.

### Western blot analysis

Proteins from BVECs were dissolved by pipetting the cells with RIPA buffer (50 mM Tris-HCl (pH 8), 150 mM NaCl, 1% NP-40, 0.5% sodium deoxycholate, 0.1% sodium dodecyl sulfate, and protease inhibitor cocktail). Their concentrations were quantified using a Bio-Rad protein assay kit (Bio-Rad, Hercules, CA, USA). Protein lysates were denatured at 70 °C for 10 min in NuPAGE LDS buffer (Thermo Fisher Scientific) and loaded on a Bolt 4–12% Bis-Tris Gel (Thermo Fisher Scientific). Proteins were transferred on nitrocellulose membranes using an iBlot dry blotting system (Thermo Fisher Scientific,). After 1 h of blocking with 5% non-fat milk (Wako) in Tris-Buffered Saline (TBS; 50 mM Tris-HCl, 138 mM NaCl, 2.7 mM KCl) and 0.1% Tween 20 (Wako), the membranes were incubated with primary antibodies overnight at 4 °C, followed by incubation with appropriate horseradish peroxidase (HRP)-conjugated secondary antibodies (anti-mouse IgG-HRP [Sigma-Aldrich] or goat anti-rabbit IgG-HRP [Santa Cruz Biotechnology, Inc., Dallas, TX, USA]) for 1 h at room temperature. The following antibodies were used for primary antibodies: rabbit anti-Caveolin-1 monoclonal antibody (D46G3, Cell Signaling Technology, Inc., Danvers, MA, USA), and mouse anti-β-actin monoclonal antibody (AC-15, Sigma-Aldrich). The membrane was visualized with ImmunoStar Basic (Wako) and imprinted onto films by an automatic processer (Eastman Kodak Company, Rochester, NY, USA).

### ChIP analysis

ChIP assays were performed using a SimpleChIP Plus Enzymatic Chromatin IP kit according to the manufacturer’s instructions (Cell Signaling Technology, Inc.). Briefly, brains were minced by scissors in PBS with protease inhibiter cocktail (Cell Signaling Technology, Inc.) and then fixed with 1.5% formaldehyde for 20 min to cross-link the proteins. To stop the cross-link, glycine was added, and the samples were subsequently processed according to the manufacturer’s instructions to obtain chromatin. The resulting chromatin was digested with micrococcal nuclease (Cell Signaling Technology, Inc.) and sonicated into DNA/protein fragments of 150–400 bp in size. The sonication was performed as three sets of 20-s pulses using a Bioruptor (Cosmo Bio Co., Ltd., Tokyo, Japan). The chromatin from the brains of the control and IAE groups were incubated with mouse anti-Di/Tri-Methyl-Histone H3 (Lys9) monoclonal antibody (6F12, Cell Signaling Technology, Inc.) or with normal rabbit IgG (Cell Signaling Technology, Inc.) as a negative control. The complexes of protein and antibody were captured with ChIP-Grade Protein G Magnetic Beads (Cell Signaling Technology, Inc.). The cross-links were then reversed, and DNA was purified following the manufacturer’s instructions. The ChIP efficiency was controlled by quantitative real-time PCR analysis with PowerUp SYBR Green Master Mix (Thermo Fisher Scientific) and the following primers: *Cav1* promoter region, 5ʹ-CAGGCTCTCAGCTCCCCGCCG-3ʹ (forward) and 5ʹ-GTATAGAGGGGGGAAAGGCGC-3ʹ (reverse) (Invitrogen, Carlsbad, CA, USA).

### Statistical analysis

Statistical differences were analysed by one-way analysis of variance (ANOVA) with Tukey’s multiple comparisons test. P values of <0.05 were considered significant. Graphs and statistical tests were made by GraphPad Prism version 7.00 (GraphPad Software, San Diego, CA, USA).

## Electronic supplementary material

Supplementary Figure
