# Supplementary material for: Abrogated Caveolin-1 expression via histone modification enzyme Setdb2 regulates brain edema in a mouse model of influenza-associated encephalopathy
Source: Sci Rep. 2019 Jan 22;9:284. doi: 10.1038/s41598-018-36489-8 (PMC6342998; doi:10.1038/s41598-018-36489-8)
Supplement: Supplementary file 1 — Supplementary Figure [file 41598_2018_36489_MOESM1_ESM.docx]

**Supplementary information**

**Abrogated Caveolin-1 expression via histone modification enzyme Setdb2 regulates brain edema in a mouse model of influenza-associated encephalopathy**

Natsuko Imakita, Masahiro Kitabatake, Noriko Ouji-Sageshima, Atsushi Hara, Shoko Morita-Takemura, Kei Kasahara, Akihiro Matsukawa, Akio Wanaka, Keiichi Mikasa, and Toshihiro Ito*

**Figure S1**

**
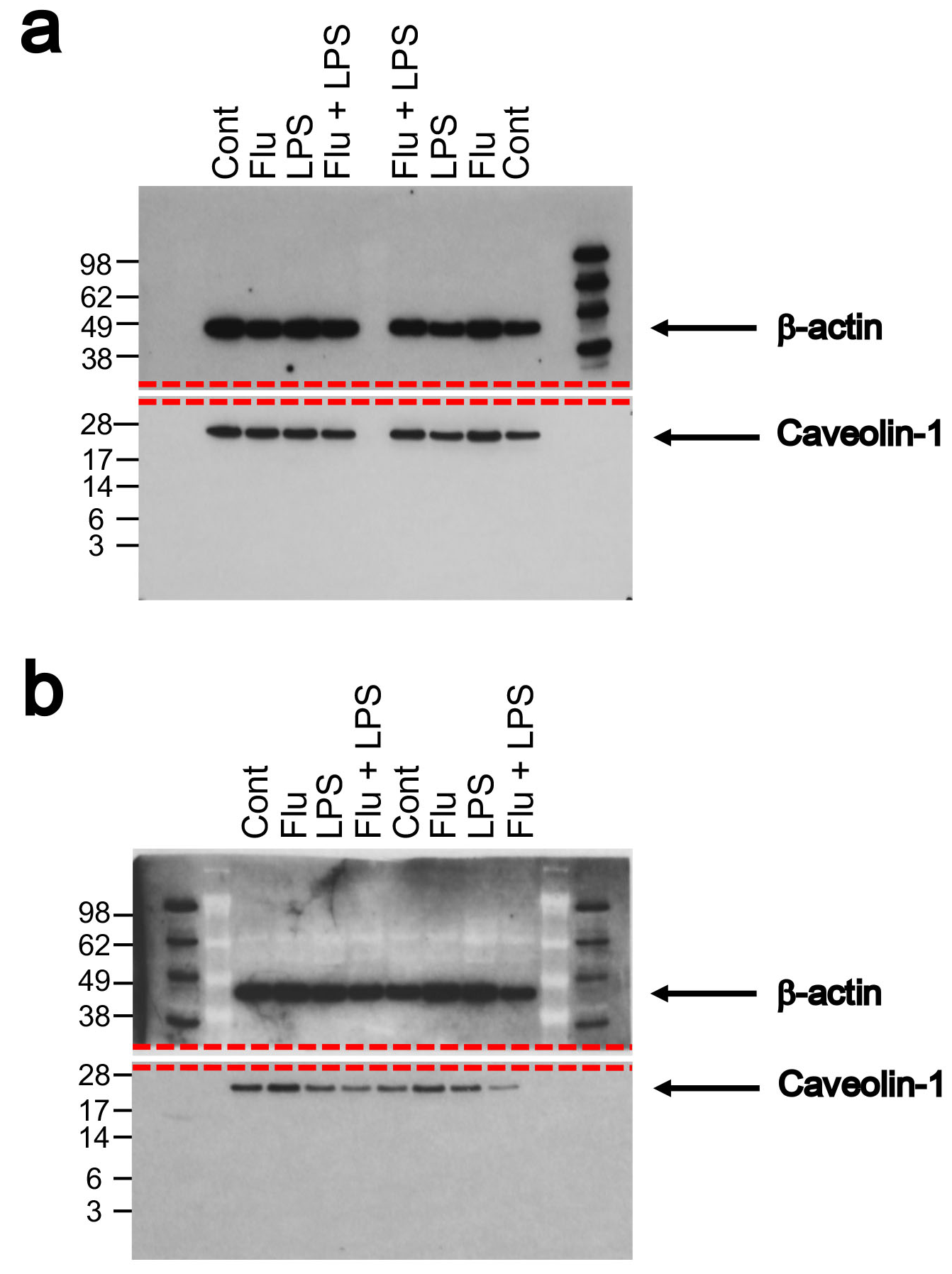
**

**Supplementary figure S1. Full-length images of figure 4b.**

Western blotting analysis of Caveolin-1 and β-actin from BVECs of the control, Flu, LPS, and IAE (Flu + LPS) groups. a) 8h after LPS administration, and b) 24h after LPS administration. Protein-transferred membrane was divided into two parts at about 30 kDa (red dot line). Upper part of the membrane was incubated with anti-β-actin antibody, and lower part of the membrane was incubated with anti-Caveolin-1 antibody.
